# Supplementary material for: AMBERff at Scale: Multimillion-Atom Simulations with AMBER Force Fields in NAMD
Source: J Chem Inf Model. 2024 Jan 4;64(2):543–54. doi: 10.1021/acs.jcim.3c01648 (PMC10806814; doi:10.1021/acs.jcim.3c01648)
Supplement: Supplementary file 1 — ci3c01648_si_001.pdf [file ci3c01648_si_001.pdf]

Supporting Information

AMBERff at scale: Multimillion-atom simulations with AMBER force fields in NAMD

Santiago Antolínez,[†] Peter Eugene Jones,[†] James C. Phillips,[‡] and Jodi A. Hadden-Perilla[*,†]

†*Department of Chemistry and Biochemistry, University of Delaware, Newark, DE 19716*

‡*National Center for Supercomputing Applications, University of Illinois at Urbana-Champaign, Urbana, IL 61801*

*Corresponding author; E-mail: jhadden@udel.edu

Tables

Table S1: Jensen-Shannon Divergence values comparing biophysical property distributions.

Property	$JSD(AMBER \| NAMD)$
Protein test case: UBQ	
RMSD	1.86×10^{-3}
RGYR	4.89×10^{-3}
SASA	1.70×10^{-3}
Nucleic acid test case: DDD	
shear	2.20×10^{-4}
stretch	4.35×10^{-4}
stagger	4.93×10^{-4}
buckle	3.03×10^{-4}
propeller	6.52×10^{-3}
opening	5.19×10^{-4}
α	6.75×10^{-4}
β	1.25×10^{-4}
γ	5.03×10^{-4}
δ	1.90×10^{-3}
χ	5.44×10^{-4}
ϵ	7.50×10^{-5}
ζ	7.50×10^{-5}
shift	2.45×10^{-4}
slide	1.29×10^{-3}
rise	5.22×10^{-3}
tilt	4.14×10^{-4}
roll	6.77×10^{-4}
twist	4.78×10^{-3}
x-displacement	3.47×10^{-4}
y-displacement	4.22×10^{-4}
helical rise	8.72×10^{-4}
inclination	9.52×10^{-4}
tip	4.48×10^{-4}
helical twist	3.17×10^{-3}
major groove	7.46×10^{-3}
minor groove	2.21×10^{-3}
Lipid test case: POPC	
APL	2.14×10^{-3}

Figures

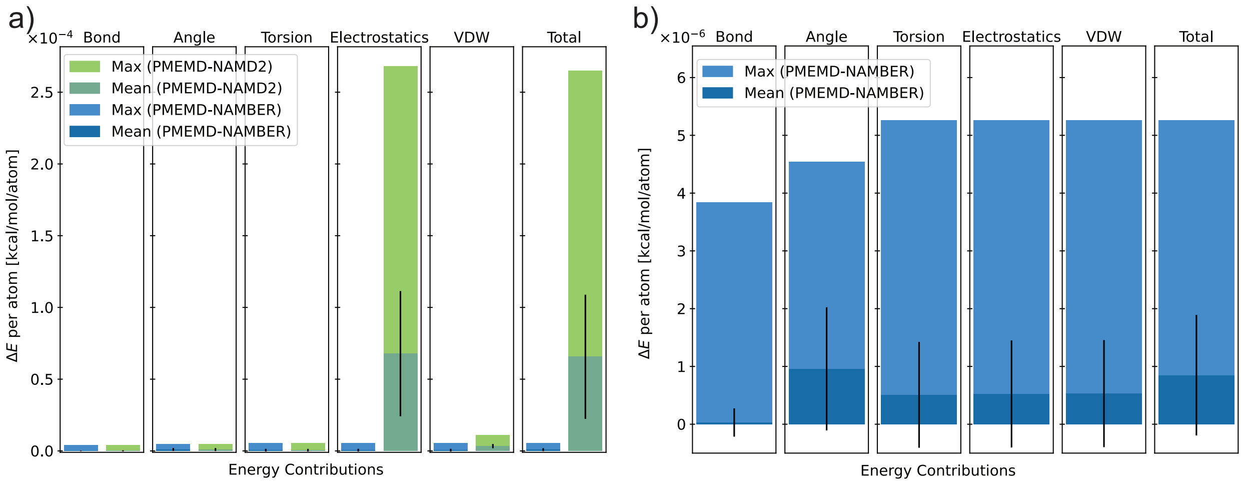

Figure S1: Comparison of single-point energies calculated with AMBER engine versus NAMD (green) and NAMBER (blue) for the ff14SB force field. **a)** Per-atom single-point energy deviations decomposed into bonds, angles, torsions, electrostatics, and van der Waals contributions. **b)** Electrostatics energy deviations decrease by two orders of magnitude when using NAMBER, a version of NAMD compiled with AMBER's Coulomb's constant. Error bars represent standard deviation.

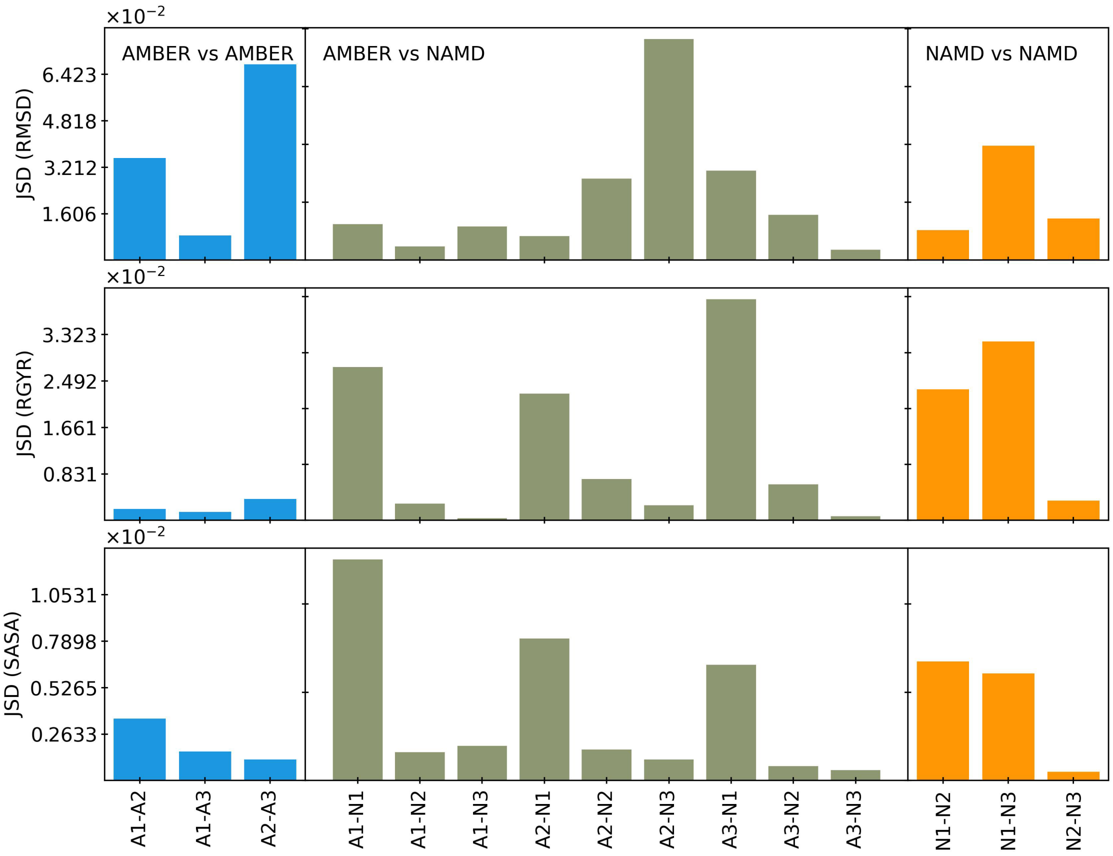

Figure S2: Jensen-Shannon Divergence values comparing biophysical property distributions for individual ubiquitin simulations.

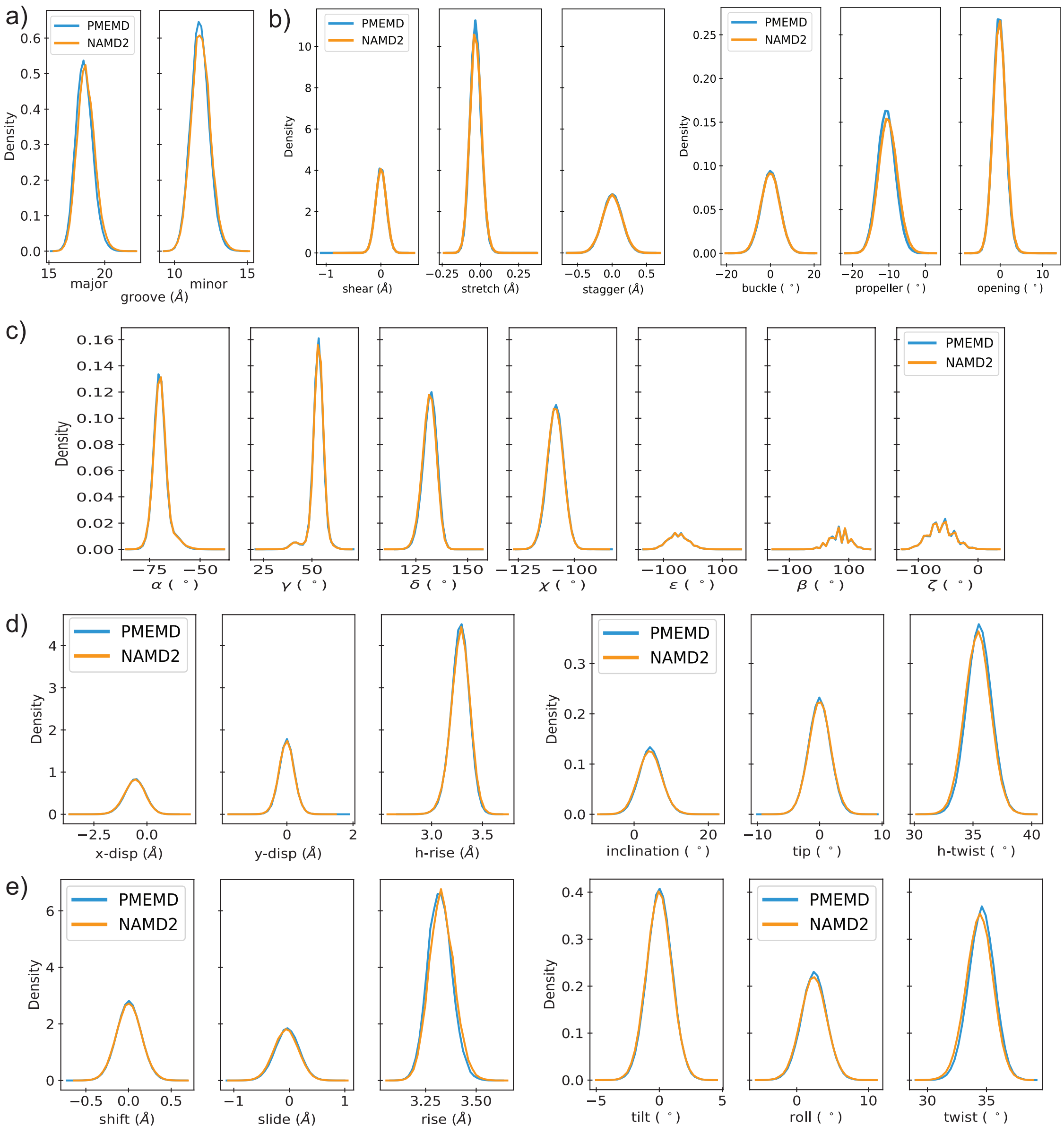

Figure S3: Distributions obtained from simulations in AMBER (blue) and NAMD (orange) for the complete set of 27 structural parameters tracked for the Dickerson-Drew dodecamer. Structural parameters include: **a)** major and minor groove widths, **b)** base pair parameters (shear, stretch, stagger, buckle, propeller and opening), **c)** backbone dihedral angles, **d)** helical base step parameters (displacement along x-axis and y-axis, helical rise, inclination, tip, and helical twist), and **e)** base step parameters (shift, slide, rise, tilt, roll, and twist).

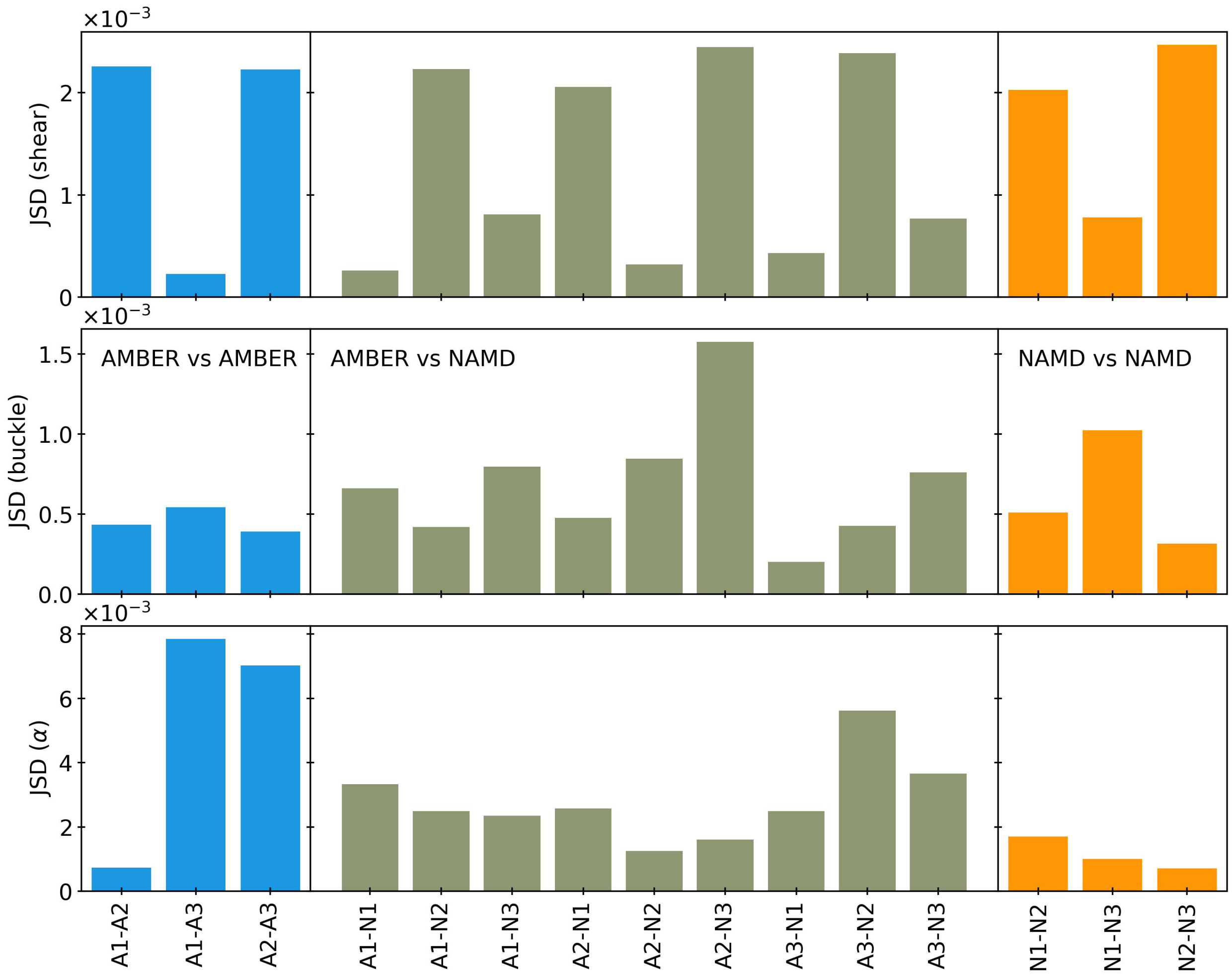

Figure S4: Jensen-Shannon Divergence values comparing biophysical property distributions for individual nucleic acid simulations.

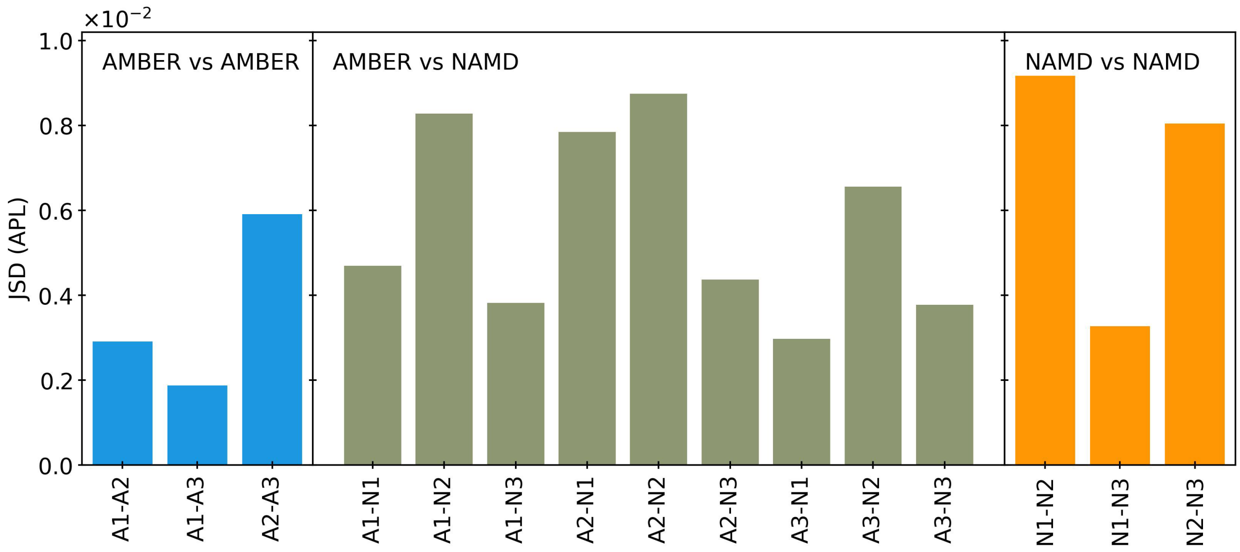

Figure S5: Jensen-Shannon Divergence values comparing biophysical property distributions for individual lipid simulations.

Scripts

Example VMD script using *psfgen*

```
#Append path to AMBER cognizant plugins
lappend auto_path /path/to/AMBERff-in-NAMD/plugins

# use PSFGEN
package require psfgen

resetpsf

set topdir /path/to/AMBERff-in-NAMD
topology $topdir/ff14SB/ff14SB.rtf
topology $topdir/solvents/opc_ions.str

# load protein pdb with AMBER atom and residue names
set molid [mol new ubq_ambernames.pdb]

# Define segments
set sel [atomselect $molid protein]
$sel set segname PRT
$sel writepdb prt.pdb
$sel delete

segment PRT {pdb prt.pdb}
coordpdb prt.pdb PRT

regenerate angles dihedrals
guesscoord
```

```
writepsf protein.psf
writepdb protein.pdb

mol delete $molid

set molid [mol new protein.psf]
mol addfile protein.pdb

set sel [atomselect $molid protein]
set charge [expr round([vecsum [$sel get charge]])]
set num [expr abs(3*$charge)]
$sel delete

# use CIONIZE
package require cionize
namespace import ::cionize::*

cionize -mol $molid -np 1 -mg -ions "{NA $num 1} {CL $num -1}"

segment NA {pdb cionize-ions_1-NA.pdb}
coordpdb cionize-ions_1-NA.pdb


segment CL {pdb cionize-ions_1-CL.pdb}
coordpdb cionize-ions_1-CL.pdb


# write psfpdb with all cionize ions
```

```
writepsf protein_cionize.psf
writepdb protein_cionize.pdb

mol delete $molid

# get rid of excess cionize ions
set molid [mol new protein_cionize.psf]
mol addfile protein_cionize.pdb

set bad [atomselect $molid "all not within 20 of protein"]

foreach seg [$bad get segid] res [$bad get resid] {
   delatom $seg $res }

writepsf protein_cionize_ions.psf
writepdb protein_cionize_ions.pdb

mol delete $molid

set name protein_cionize_ions
set molid [mol new $name.psf ]
mol addfile $name.pdb

# find box dimensions with a 15A padding
set bsize 15
set model [atomselect $molid protein]
set center [measure center $model]
set dims [vecsub [lindex [measure minmax $model] 1] [lindex [measure minmax\
$model] 0 ]]
```

```
set maxv [expr max([lindex $dims 0],[lindex $dims 1], [lindex $dims 2])]
$model delete

set blen [expr ($maxv+2*$bsize)/2]
set bmin {}
set bmax {}
foreach i [lindex $center] {lappend bmin [expr $i-$blen]}
foreach i [lindex $center] {lappend bmax [expr $i+$blen]}

# load SOLVATE 1.8 and AUTOIONIZE 1.6 (both suppport AMBER solvents)
package require solvate 1.8
package require autoionize 1.6

mol delete $molid
# Solvate with OPC water model using -ambersolvent flag
solvate $name.psf $name.pdb -minmax [list $bmin $bmax] -o ${name}_solvent\
-ambersolvent opc


# Autoionize with AMBER ions NA and CL using the -amber flag
autoionize -psf ${name}_solvent.psf -pdb ${name}_solvent.pdb -cation NA -anion\
CL -sc 0.150 -o ${name}_solvent_sc150mM -seg I1 -amber


exit
```

PMRTOP in AMBER: Single point energy configuration file

```
&cntrl

ntx=1, irest=0,

ntc=1, ntf=1,

nstlim=0, dt=0.002,

ntpr=1, ntwx=0, ntwr=0,

cut=999.0, fswitch=0,

ntt=0, ntb=0, ntp=0,

ioutfm=1, ntave=1000,

jfastw=0,

/

&ewald

ew_type=0,

vdwmeth=0,

eedmeth=1,

/
```

PSF in NAMD: Single point energy configuration file

```
structure     psfgen.psf
coordinates   psfgen.pdb
#
source         $ffdir/$NAMDRC
restartfreq       1
dcdfreq           1
xstFreq           1
outputEnergies    1
outputPressure    0
outputname        nve
amber         off
rigidBonds    None
cutoff        999.0
pairlistdist  1000.0
switching     off
exclude       scaled1-4
readexclusions yes
1-4scaling    0.83333333
#
timestep          2.00
nonbondedFreq     1
fullElectFrequency 1
langevin          off
langevinPiston    off
PME               off
PMETolerance      1.0e-6
PMEInterpOrder    4
FFTWUseWisdom     no
```

```
PMEGridSizeX      48
PMEGridSizeY      48
PMEGridSizeZ      48
binaryOutput      off
run               0
```

Protein case study: NAMD configuration file

```
############################
## ADJUSTABLE PARAMETERS ##
############################
# Define structure and coordinate files
structure             /path/to/structurefile.psf
coordinates           /path/to/coordinatefile.pdb
# Simulation is a restart
# Define restart files for system coordinates and velocities
binCoordinates        /path/to/previous_step.restart.coor
binVelocities         /path/to/previous_step.restart.vel
extendedSystem        /path/to/previous_step.restart.xsc
# Define variables which will be used later in the script
set paramdir          /path/to/AMBERff-in-NAMD
set temperature       310

############################
## SIMULATION PARAMETERS ##
############################
## Force Field Input
source                $paramdir/ff14SB/namdrc.ff14SB
source                $paramdir/solvents/namdrc.tip3p
## Force Field Parameters
exclude               scaled1-4 ; # Exclude 1-2s and 1-3s, scale 1-4s
1-4scaling            0.83333333 ; # scee AMBER
cutoff                8.0 ; #
pairlistdist          10.0 ; # Pairlist search radius
switching             off ; #
zeromomentum          on ; #
```

```
# Analytical correction to account for neglected long-range VDW interactions
ljcorrection          on ;
## Integrator Parameters
timestep              2.0 ; # dt
rigidBonds            all ; # ntc=2/ntf=2, SHAKE bonds to H
rigidTolerance        1.0e-8 ;
useSettle             on ; # SETTLE for waters
nonbondedFreq         1 ; # Frequency to calculate nonbonded interactions
fullElectFrequency    2 ; # Frequency to calculate full electrostatics
longSplitting         c2 ; # Long/short range splitting method
stepspercycle         20 ; # nsnb, Pairlist update
## Periodic Boundary Conditions (ntb>0)
wrapAll               on ; # iwrap=1
## PME (for full-system periodic electrostatics)
PME                   yes ;   # ntb>0/&ewald
PMEInterpOrder        4 ;     # Cubic Interpolation
PMEGridSpacing        1.0 ;   # Angstroms
## Constant Temperature Control (ntt>0)
langevin              on ; # ntt=3, Langevin Thermostat
langevinTemp          $temperature ; # temp0
langevinDamping       1.0 ; # gamma_ln
langevinHydrogen      off ; # Use with SHAKE
## Constant Pressure Control (ntb=2/ntp>0)
useGroupPressure      yes ; # Required for SHAKE
useFlexibleCell       no ; # ntp=1, Isotropic pressure scaling
useConstantRatio      no ; #
BerendsenPressure     on ; # barostat=1
BerendsenPressureTarget 1.0 ; # target pressure in bar
BerendsenPressureCompressibility    4.46e-5 ; #
```

```
BerendsenPressureRelaxationTime     100 ; #
BerendsenPressureFreq 10 ; #
## Harmonic restraints
constraints           off ; # ntr=0
## Output
outputName            $outputname ; #
binaryOutput          yes ; # ioutfm=1
restartfreq           10000 ; # ntwr, restart
dcdfreq               5000 ; # ntwx, trajectory
xstFreq               5000 ; # ntwx, xst trajectory
outputEnergies        5000 ; # ntpr, mdout
outputPressure        5000 ; # ntpr, mdout

######################
## EXECUTION SCRIPT ##
######################
run 250000000 ; # Run 500 ns
```

Lipid case study: NAMD configuration file

```
############################
## ADJUSTABLE PARAMETERS ##
############################
# Define structure and coordinate files
structure             /path/to/structurefile.psf
coordinates           /path/to/coordinatefile.pdb
# Simulation is a restart
# Define restart files for system coordinates and velocities
binCoordinates        /path/to/previous_step.restart.coor
binVelocities         /path/to/previous_step.restart.vel
extendedSystem        /path/to/previous_step.restart.xsc
# Define variables which will be used later in the script
set paramdir          /path/to/AMBERff-in-NAMD
set temperature       303

############################
## SIMULATION PARAMETERS ##
############################
## Force Field Input
source               $paramdir/lipid21/namdrc.lipid21
source               $paramdir/solvents/namdrc.tip3p
## Force Field Parameters
exclude               scaled1-4 ; # Exclude 1-2s and 1-3s, scale 1-4s
1-4scaling            0.83333333 ; # scee AMBER
cutoff                8.0 ; #
pairlistdist          10.0 ; # Pairlist search radius
switching             off ; #
zeromomentum          on ; #
```

```
# Analytical correction to account for neglected long-range VDW interactions
ljcorrection          on ; #
## Integrator Parameters
timestep              2.0 ; # dt
rigidBonds            all ; # ntc=2/ntf=2, SHAKE bonds to H
rigidTolerance        1.0e-8 ; #
useSettle             on ; # SETTLE for waters
nonbondedFreq         1 ; # Frequency to calculate nonbonded interactions
fullElectFrequency    2 ; # Frequency to calculate full electrostatics
longSplitting         c2 ; # Long/short range splitting method
stepspercycle         20 ; # nsnb, Pairlist update
## Periodic Boundary Conditions (ntb>0)
wrapAll               on ; # iwrap=1
## PME (for full-system periodic electrostatics)
PME                   yes ;  # ntb>0/&ewald
PMEInterpOrder        4 ;    # Cubic Interpolation
PMEGridSpacing        1.0 ;  # Angstroms
## Constant Temperature Control (ntt>0)
langevin              on ; # ntt=3, Langevin Thermostat
langevinTemp          $temperature ; # temp0
langevinDamping       1.0 ; # gamma_ln
langevinHydrogen      off ; # Use with SHAKE
## Constant Pressure Control (ntb=2/ntp>0)
useGroupPressure      yes ; # Required for SHAKE
useFlexibleCell       yes ; #
useConstantRatio      yes ; # ntp=2, Semi-isotropic pressure scaling
BerendsenPressure     on ; # barostat=1
BerendsenPressureTarget 1.0 ; # target pressure in bar
BerendsenPressureCompressibility    4.46e-5 ; #
```

```
BerendsenPressureRelaxationTime     100 ; #

BerendsenPressureFreq 10 ; #

## Harmonic restraints

constraints          off ; # ntr=0

## Output

outputName           $outputname ; #

binaryOutput         yes ; # ioutfm=1

restartfreq          10000 ; # ntwr, restart

dcdfreq              5000 ; # ntwx, trajectory

xstFreq              5000 ; # ntwx, xst trajectory

outputEnergies       5000 ; # ntpr, mdout

outputPressure       5000 ; # ntpr, mdout


######################

## EXECUTION SCRIPT ##

######################

run 250000000 ; # Run 500 ns
```

Nucleic acid case study: NAMD configuration file

```
############################
## ADJUSTABLE PARAMETERS ##
############################
# Define structure and coordinate files
structure           /path/to/structurefile.psf
coordinates         /path/to/coordinatefile.pdb
# Simulation is a restart
# Define restart files for system coordinates and velocities
binCoordinates      /path/to/previous_step.restart.coor
binVelocities       /path/to/previous_step.restart.vel
extendedSystem      /path/to/previous_step.restart.xsc
# Define variables which will be used later in the script
set paramdir        /path/to/AMBERff-in-NAMD
set temperature     310

############################
## SIMULATION PARAMETERS ##
############################
## Force Field Input
source              $paramdir/OL15/namdrc.OL15
source              $paramdir/solvents/namdrc.tip3p
## Force Field Parameters
exclude             scaled1-4 ; # Exclude 1-2s and 1-3s, scale 1-4s
1-4scaling          0.83333333 ; # scee AMBER
cutoff              8.0 ; #
pairlistdist        10.0 ; # Pairlist search radius
switching           off ; #
zeromomentum        on ; #
```

```
# Analytical correction to account for neglected long-range VDW interactions
ljcorrection          on ; #
## Integrator Parameters
timestep              2.0 ; # dt
rigidBonds            all ; # ntc=2/ntf=2, SHAKE bonds to H
rigidTolerance        1.0e-8 ; #
useSettle             on ; # SETTLE for waters
nonbondedFreq         1 ; # Frequency to calculate nonbonded interactions
fullElectFrequency    2 ; # Frequency to calculate full electrostatics
longSplitting         c2 ; # Long/short range splitting method
stepspercycle         20 ; # nsnb, Pairlist update
## Periodic Boundary Conditions (ntb>0)
wrapAll               on ; # iwrap=1
## PME (for full-system periodic electrostatics)
PME                   yes ;   # ntb>0/&ewald
PMEInterpOrder        4 ;     # Cubic Interpolation
PMEGridSpacing        1.0 ;   # Angstroms
## Constant Temperature Control (ntt>0)
langevin              on ; # ntt=3, Langevin Thermostat
langevinTemp          $temperature ; # temp0
langevinDamping       1.0 ; # gamma_ln
langevinHydrogen      off ; # Use with SHAKE
## Constant Pressure Control (ntb=2/ntp>0)
useGroupPressure      yes ; # Required for SHAKE
useFlexibleCell       no ; # ntp=1, Isotropic pressure scaling
useConstantRatio      no ; #
BerendsenPressure     on ; # barostat=1
BerendsenPressureTarget 1.0 ; # target pressure in bar
BerendsenPressureCompressibility    4.46e-5 ; #
```

```
BerendsenPressureRelaxationTime     100 ; #
BerendsenPressureFreq 10 ; #
## Harmonic restraints
constraints           off ; # ntr=0
## Output
outputName            $outputname ; #
binaryOutput          yes ; # ioutfm=1
restartfreq           20000 ; # ntwr, restart
dcdfreq               10000 ; # ntwx, trajectory
xstFreq               10000 ; # ntwx, xst trajectory
outputEnergies        5000 ; # ntpr, mdout
outputPressure        5000 ; # ntpr, mdout

######################
## EXECUTION SCRIPT ##
######################
run 250000000 ; # Run 500 ns
```
